# Effect of ZnO Nanoparticles on *Brassica nigra* Seedlings and Stem Explants: Growth Dynamics and Antioxidative Response

**DOI:** 10.3389/fpls.2016.00535

**Published:** 2016-04-20

**Authors:** Hira Zafar, Attarad Ali, Joham S. Ali, Ihsan U. Haq, Muhammad Zia

**Affiliations:** ^1^Department of Biotechnology, Quaid-i-Azam UniversityIslamabad, Pakistan; ^2^Department of Pharmacy, Quaid-i-Azam UniversityIslamabad, Pakistan

**Keywords:** antioxidative activities, *Brassica nigra*, callus, nanoparticles, rooting, shooting, ZnO

## Abstract

Nanoparticles (NPs) have diverse properties when compared to respective chemicals due to their structure, surface to volume ratio, morphology, and reactivity. Toxicological effects of metallic NPs on organisms including plants have been reported. However, to the best of our knowledge, there is still not any report on the effect of NPs on *in vitro* culture of plant explants. In this study, ZnO NPs concentration ranging from 500 to 1500 mg/L adversely affects the *Brassica nigra* seed germination and seedling growth and also lead to an increase in the antioxidative activities and non-enzymatic antioxidants. While, culturing the stem explants of *B. nigra* on Murashige and Skoog (MS) medium at lower concentration of ZnO NPs (1–20 mg/L) resulted in the production of white thin roots with thick root hairs. At 10 mg/L ZnO NPs, shoots emergence is also observed. The developed calli/roots showed 79% DPPH (2,2-diphenyl-1-picryl hydrazyl) radical scavenging activity at 10 mg/L. The total antioxidant and reducing power potential also significantly affected in presence of ZnO NPs. Moreover, an increase in non-enzymatic antioxidative molecules, phenolics (up to 0.15 μg GAE/mg FW) and flavonoids (up to 0.22 μg QE/mg FW), depending on NPs concentration is also observed. We conclude that ZnO NPs may induce roots from explants cultured on appropriate medium that can be used for production of valuable secondary metabolites.

## Introduction

Metallic nanoparticles (NPs) are now frequently used in industries for variety of applications. However, their release in environment makes them a threatening agent to living organisms in a way of toxicity. The toxic effects are due to size, surface area ratio, morphology, nature, composition, reactivity, and others (Zaka et al., 2016). Although NPs of toxic elements are supposed to be more toxic but less or least toxic elements are also proven toxic when switched to NPs. Engineering methodology and post processing of NPs alter the physico-chemical properties and reactivity. The toxicity of metal oxide NPs involves three distinct mechanisms; (i) release of respective metal ion; (ii) surface modification and interaction with media; (iii) disruption biomolecules (Brunner et al., 2006).

Among various metals, zinc (Zn) plays vital role in biochemical, physiological and anatomical responses but below to threshold level. Zinc oxide (ZnO) NPs are widely used in paints, coating materials, medical and personal care products, and many more. ZnO NPs are also used as UV protector and absorber material. Beside their potential use, it has increased environmental and health risks due to their interaction with biological and chemical materials (Chithrani et al., 2006). The production of ZnO NPs is up to 528 tons/year and there is increase in production and utilization with time (Zhang and Saebfar, 2010). Increased use in industrial products has led their release in environment, i.e., from cosmetic products, sunscreens and others (Danovaro et al., 2008). There are limited studies reported on phytotoxicity of ZnO nanoparticles on plants. Some plant species, i.e., rape, corn, lettuce, radish, ryegrass, cucumber (Lin and Xing, 2007), zucchini (Stampoulis et al., 2009), garden cress, and broad bean (Manzo et al., 2011), and wheat (Du et al., 2011) are sensitive toward ZnO NPs. Presence of ZnO NPs in surrounding environment affect plant architecture, physiology and biochemistry. The toxicity is considered due to internalization of NPs, accumulation in root tissue and root surface, dissolution of zinc ions from NPs along with other physio chemical properties (Ma et al., 2009, 2011).

*Brassica* species are wild and also grown as food and fodder crops. *Brassica nigra* is commonly grown for oil extraction, animal cake production, and green manure. It is considered tolerant to heavy metals (Angelova and Ivanov, 2009) and also known as metal accumulators and potential phytoextraction (Van Ginneken et al., 2007). Based on resistivity and tolerance toward nanoparticles, *B. nigra* could be considered as a model plant to study reaction mechanisms of metallic nanoparticles on plant growth.

From an ecological perspective, understanding the ZnO NPs toxicity toward environmentally relevant plant species is of great importance. The aim of this study is to determine the toxicity of ZnO nanoparticles on *B. nigra* including its seed germination; seedling growth and antioxidative response. Moreover, stem explants cultured in the presence of ZnO NPs in MS medium is used to determine the response of undifferentiated mass of cells. We believe that the experimental results would contribute to the enrichment of knowledge pertaining to the interactions of ZnO NPs with plant system.

## Materials and Methods

### Materials

All the chemicals and reagents were purchased from Sigma–Aldrich and Merck. MS media was acquired from Phytotech. ZnO NPs were synthesized by co-precipitation method (reported elsewhere). The NP diameter (<100 nm) is determined through scanning electron microscopy.

### Effect of ZnO NPs on *B. nigra* Seed Germination and Growth

#### Preparation of ZnO Supplemented Media

To investigate toxicity of ZnO NPs, plain agar media was used. Briefly, ZnO NPs were suspended at 0 (control), 500, 1000, and 1500 mg/L in distilled autoclaved water. Sucrose was added at 3% as carbon source. To avoid aggregation of NPs, the medium was sonicated for 30 min. The pH was adjusted to 5.8 and agar (0.7%) was added as solidifying agent. Agar was dissolved by heating. The medium was dispensed as 30 ml/100 ml in a conical flask after thorough shaking. The media was autoclaved at 121°C, 15 psi pressure for 20 min. To avoid agglomeration or settling of NPs at the base, the medium was allowed to cool up to 45°C; shacked well and the flasks were kept at -4°C till solidification.

#### Seed Inoculation and Growth Observation

*Brassica nigra* seeds were obtained from National Agriculture Research Council (NARC) Islamabad Pakistan with germination efficiency >98%. Under aseptic conditions, the seeds were treated with 0.1% mercuric chloride (w/v) for 2–3 min, followed by thorough rinsing with sterilized distilled water. Four seeds were inoculated in each flask and five flasks for each concentration were prepared. The flasks were kept in growth chamber at 25°C in dark. The germination efficiency was observed at 5th day of seed inoculation thereafter the flasks were transferred to 16/8 light dark cycle for additional 7 days. At the end of experiment plants were isolated and analyzed for shoot, root length, and fresh weight (FW). The plant parts were dried under vacuum at 45°C for 48 h to calculate dry biomass.

### Effect of ZnO NPs on Stem Explant Culture

#### Media Preparation

Murashige and Skoog (1962) media was used as basal medium. ZnO NPs (0, 1, 5, 10, and 20 mg/l) were supplemented in MS media along with 3% sucrose. pH was adjusted at 5.8 and media was sonicated for 30 min to avoid aggregation of NPs. Agar (0.7%) was added and dissolved by heating. The media was dispensed as 30 ml/100 ml in a conical flask with thorough shaking. Then autoclaved at 121°C, 15 psi pressure for 20 min. To avoid agglomeration or settling of NPs at the base, the media was allowed to cool up to 45°C; shacked well and flasks were kept at -4°C till solidification.

#### Explant Source and Growth Conditions

The 14 days old *B. nigra* plants grown on plain agar medium were used as explants source. Under aseptic conditions, stem was separated and cut into 8 mm pieces as explants and inoculated on the surface of media. The flasks were incubated at 25°C and 16/8 light dark period in growth room. The test was performed in triplicate; each replicate contained 10 explants. Data were recorded after 30 days of inoculation and FW and dry weight (DW) were measured.

### Preparation of Extracts and Determination of Antioxidative Potential

The dried plant materials were ground in mortar and pestle and suspension was prepared in DMSO at 100 mg/ml in eppendorf tubes. The tubes were kept at room temperature for 48 h and centrifuged at 5000 rpm for 3 min. The supernatant was used for estimation of antioxidative activities (DPPH based free radical scavenging activity, total antioxidant potential, total reducing power) and non-enzymatic antioxidants (phenolics and flavonoids).

#### Determination of Free Radical Scavenging Potential

2,2-diphenyl-1-picryl hydrazyl (DPPH) reagent was employed for the determination of free radical scavenging activity (Rehman et al., 2014). Briefly, 10 μL of extract was mixed with 190 μL of DPPH (0.004% w/v in methanol). The reaction mixture was incubated in dark for 1 h. The optical density was measured at 515 nm using microplate reader. Ascorbic acid was employed as positive standard, while DMSO as negative control. Percent inhibition was calculated by the following formula:

Percent inhibition of the test sample=% scavenging activity=(1−Abs/Abc) × 100.

Where Ab_s_ is the absorbance of DPPH solution with sample, and Ab_c_ indicates the absorbance of negative control (containing the reagent and solvent only).

#### Determination of Total Antioxidant Capacity (TAC)

Total antioxidant activity of extracts was evaluated by method modified by Fatima et al. (2015). An aliquot of 100 μL from stock solution of each extract was mixed with 900 μL reagent solutions comprising of 0.6 M sulfuric acid, 4 mM ammonium molybdate and 28 mM sodium phosphate. The reaction mixtures were incubated at 95°C for 90 min followed by cooling at room temperature and absorbance was measured at 695 nm by using microplate reader. DMSO (100 μL) in place of test samples was used as control. For calibration curve, ascorbic acid was used as positive control. The resultant TAC was expressed as μg ascorbic acid equivalent per mg FW (μg AAE/mg FW).

#### Determination of Total Reducing Power (TRP)

The reduction potential was investigated according to previously described procedure (Haq et al., 2010). Briefly, 100 μL of each sample was mixed with 200 μL of phosphate buffer (0.2 M, pH 6.6) and 250 μL of 1% w/v potassium ferricyanide. The resulting mixture was incubated for 20 min at 50°C. After that, the reaction was acidified with 200 μL of 10% w/v trichloroacetic acid. The resultant mixtures were centrifuged at 3000 rpm for 10 min and supernatant layer (150 μL) was mixed with 50 μL of 0.1% w/v ferric chloride solution and optical density was measured at 630 nm. Ascorbic acid was maintained as positive control and results were expressed as μg ascorbic acid equivalent per mg FW (μg AAE/mg FW).

#### Determination of Total Phenolic Contents (TPC)

The total phenolic contents were determined using standard protocol (Haq et al., 2010). In brief, 20 μl of extract prepared in DMSO was transferred to each well of 96 well plate. Ninety microliter of Folin–Ciocalteu reagent was added to each well and after five min 90 μl Na_2_CO_3_ (7.5% w/v in H_2_O) was mixed in each well. The reaction mixtures were incubated for 1 h and absorbance was measured at 650 nm by using microplate reader (Bioteck, USA). Blank (DMSO) and standard (gallic acid in DMSO) were run simultaneously as control. A calibration curve (*y* = 0.0135x + 0.0846, *R*^2^ = 0.986) was obtained in parallel under the same operating conditions using gallic acid (6.25–50 μg/mL). The resultant TPC was determined as μg gallic acid equivalent per mg FW (μg GAE/mg FW).

#### Determination of Total Flavonoid Content (TFC)

Total flavonoid contents were determined according to the method previously described by Haq et al. (2010). Aliquot of each extract (20 μL) was mixed with 10 μL of aluminum chloride (10% w/v) and 10 μL of potassium acetate (1 M) solutions. Thereafter distilled water was added to get a final volume of 200 μL. After 30 min of incubation; absorbance was measured by using microplate reader (Bioteck, USA) at 415 nm. The calibration curve (*y* = 0.0269x + 0.00765, *R*^2^ = 0.998) was drawn by using quercetin as standard at 0 to 40 μg/mL and the flavonoid content were established in μg quercetin equivalent per mg FW (μg QE/mg FW).

### Statistical Analysis

To investigate effect of NPs on *B. nigra* seed germination and plant characters; five flasks of each concentration were inoculated, each containing four seeds. To analyze explant response, 30 stem explants were cultured on MS medium. The results are presented as mean with standard error. All anti-oxidative tests were performed in triplicate. The means were further analyzed using analysis of variance (ANOVA) and least significant difference (LSD).

## Results

### Effect of ZnO NPs on *B. nigra* Growth and Antioxidative Response

**FIGURE 1:**
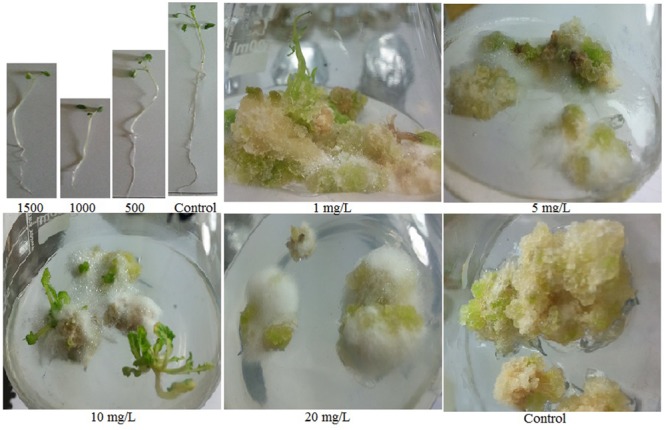
**Effect of ZnO NPs on seed germination and stem explants of *Brassica Nigra***.

The presence of ZnO NPs in the culture media significantly inhibited germination of *B*. *nigra* seeds. Increase of NPs concentration decreased germination efficiency recorded on 5th day of seed inoculation (**Table 1**). ZnO NPs in the culture media generated stimulatory effect on shoot growth but inhibited root length. At 500 mg/L ZnO, 64% increase in shoot length was observed compared to control, while at the same concentration 61% reduction in root length was observed (**Table 1**; **Figure 1**). Although, there was positive effect on shoot lengths but shoot FW decreased by increasing the NPs in the media, due to reduction in diameter of stem. At 1500 mg/L NPs, 21% reduction in shoot FW was observed. Comparatively root FW significantly decreased; 73% at 500 mg/L and 87% at 1500 mg/L. The shoot DW did not show any significant change at 500 and 1000 mg/L but at 1500 mg/L boost (1.18 mg; 51% increase in comparison with control) was observed.

**Table 1:** Effect of CuO NPs on *Brassica nigra* seed germination, plantlet length and fresh and dry weight at 5th day of seed inoculation.

Conc.	Seed germination.	Length (cm)		Fresh weight (g)		Dry weight (g)	
		Shoot	Root	Shoot	Root	Shoot	Root
Control	20	3.07 ± 0.14^d^	7.81 ± 0.56^a^	34.95 ± 3.9^a^	4.74 ± 0.23^a^	0.78 ± 0.1^c^	0.44 ± 0.02^a^
500	19	5.05 ± 0.2^a^	3.03 ± 0.2^b^	33.58 ± 2.4^ab^	1.25 ± 0.14^b^	0.84 ± 0.1^b^	0.17 ± 0.01^b^
1000	16	4.27 ± 0.11^b^	2.11 ± 0.18^c^	29.31 ± 2.8^b^	0.68 ± 0.1^c^	0.86 ± 0.1^b^	0.16 ± 0.03^b^
1500	14	3.96 ± 0.1^c^	1.91 ± 0.1^d^	27.29 ± 3.5^b^	0.59 ± 0.1^d^	1.18 ± 0.1^a^	0.11 ± 0.04^c^

*Alphabets on values represent LSD values at *p* < 0.05.*

**FIGURE 2:**
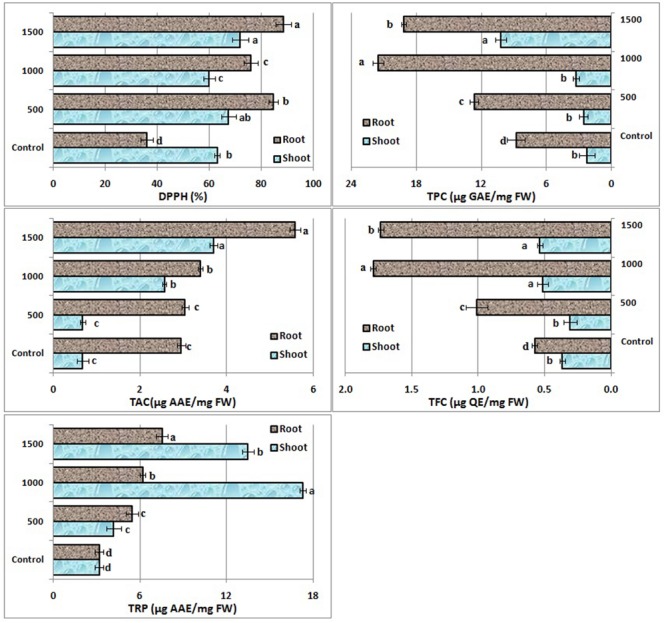
**Effect of ZnO NPs on antioxidative response of *B. nigra* seedlings.** Alphabets on bars represent least significant difference (LSD) values at *p* < 0.05.

2,2-diphenyl-1-picryl hydrazyl radical scavenging activity, total antioxidant potential, reducing power potential, total phenolic and flavonoid contents significantly varied in *B. nigra* shoots and roots on ZnO NPs exposure (**Figure 2**). Free radical scavenging activity increased 133 and 6% in root and shoot, respectively, at 500 mg/L then decreased at 1000 mg/L and again a boost was observed at 1500 mg/L. A steady increase in TAC activity was observed both for root and shoots parts. No difference was recorded at 500 mg/L compared to control, however, 88% increase in root and 436% increase in shoot TAC was observed at 1500 mg/L. Significant change in total reducing power potential (TRP) was also observed in roots and shoots (**Figure 2**). Consistent increase in TRP was recorded in roots with increase of NPs concentration in the media. However, in shoot maximum TRP was recorded at 1000 mg/L (442% increase). There was significant variation in TPC in roots. In case of shoots non-significant variation was observed at 500 and 1000 mg/L. TFC also significantly varied in roots, however, among shoot TFC non-significant variation was observed at 1000 and 1500 mg/L concentrations.

### Effect of ZnO NPs on Stem Explant Growth and Antioxidative Response

Stem explants significantly responded at different concentrations of ZnO NPs incorporated MS medium. Calli induction on hormone free MS media is normal phenomenon as multiplication of cells; the undifferentiated mass. Incorporation of ZnO NPs in MS media resulted in the emergence of minor roots along with calli, however, root induction response enhanced by increase of ZnO NPs (**Figure 1**). The roots are white, thin and covered by thick root hairs. Further it was also observed that the calli mass is decreased by increasing the ZnO concentration. At 10 mg/L ZnO, few shoots also emerged from calli; however, it looks like leaf induction. The differentiation of cells into organs and decrease in mass of calli by increasing ZnO in media resulted in the decrease of FW and DW. Stem explants produced calli on MS medium with FW 49.51 mg and DW 0.83 mg (**Table 2**). However, when ZnO was also applied in the medium, FW decreased up to 11.95 mg (75 times reduction) at 1 mg/L. The reduction gradually increased when concentration of ZnO increased. Same behavior was also observed for DW although DW reduction is less. Only 19% reduction was observed at 1 mg/L and it increased up to 63% at 20 mg/L. Significant variation was also observed in oxidative response (DPPH, TAC, and TRP) and total phenolic and flavonoids (**Table 2**). Elevated DPPH activity was calculated at all concentrations. At 1 mg/L 73% activity was calculated that reduced at 5 mg/L and again increased at 10 mg/L. A gradual increase in TAC was observed upto 10 mg/L but at 20 mg/L TAC reduced at 0.27 μg AAE/mg FW. Variation in TRP was observed at different concentrations of ZnO but all above the control value. Significant variation was also observed in TFC as compared with control but non-significant among the treatments. TPC variably responded in the range of 0.10–0.16 μg GAE/mg FW but all above the control value (0.03 μg GAE/mg FW).

**Table 2:** Effect of ZnO NPs on *Brassica nigra* stem explants response.

Conc. (mg/L)	Fresh weight (g)	Dry weight (g)	DPPH (%)	TAC (μg GAE/mg FW)	TRP (μg AAE/mg FW)	TFC(μg QE/mg FW)	TPC (μg GAE/mg FW)
Control	49.51 ± 3.8	0.83 ± 0.21^a^	28.10 ± 1^e^	0.08 ± 0.01^d^	0.07 ± 0.01^e^	0.02 ± 0.0^d^	0.03 ± 0.0^e^
1	11.95 ± 1.7^a^	0.70 ± 0.2^b^	73.44 ± 1.9^b^	0.27 ± 0.07^a^	0.79 ± 0.13^a^	0.18 ± 0.01^c^	0.16 ± 0.0^a^
5	9.87 ± 1.9^b^	0.63 ± 0.17^c^	34.26 ± 1.2^d^	0.33 ± 0.09^c^	0.26 ± 0.08^d^	0.21 ± 0.01^b^	0.10 ± 0.0^d^
10	7.51 ± 1.2^c^	0.38 ± 0.11^d^	79.02 ± 2.4^a^	0.53 ± 0.1^b^	0.72 ± 0.24^b^	0.22 ± 0.01^a^	0.14 ± 0.0^c^
20	6.37 ± 1.1^d^	0.32 ± 0.11^d^	42.06 ± 2.1^c^	0.27 ± 0.03^a^	0.58 ± 0.2^c^	0.21 ± 0.01^b^	0.15 ± 0.0^b^

*Alphabets on values represent LSD values at *p* < 0.05.*

## Discussion

### Effect of ZnO NPs on *B. nigra* Growth and Antioxidative Response

The relative germination index is extensively used as an indicator of phytotoxicity and root growth is highly sensitive biomarker for phytotoxicity assay (Ali et al., 2015). *B. nigra* seeds germinated in presence of ZnO nanoparticles (NPs) showed less germination efficiency but concentration dependent. The inhibition of seed germination might be due to penetration of nano sized particles, aggregation of NPs to micron size, and also dissolution of Zn^+2^ from ZnO (Lee et al., 2010). Nutrients along with water might also transports NPs and ions to the intracellular space of seed coat and affects seed germination (Van Dongen et al., 2003). Later direct contact of plumule or root with NPs in the media limits growth of root (Wierzbicka and Obidzinska, 1998). The inhibitory effect also depends upon permeability of seed coat for NPs; internalization in root tissues, cytotoxic and genotoxic approaches; and concentration dependency (Kumari et al., 2009, 2011; Ma et al., 2010; Nair et al., 2010).

The increase in shoot length in response of NPs might be nutritional behavior of particles or of dissociated ions but at non-lethal concentration. While, roots are in direct contact with NPs and accumulation in the root tissue or on root surface is cause of shorter root length. The presence of NPs in the agar media also produces negative effect on root elongation because agar media is non-porous, less dissolved oxygen, water logging due to NPs (Langerud and Sandvik, 1987). Lin and Xing (2007) and Manzo et al. (2011) also reported same results for ryegrass and broad bean at higher concentration (2000 mg/L) while for zucchini no negative effects were observed (Stampoulis et al., 2009). It is presumed that NPs interfere at mitotic cellular division by blocking initiation of prophase (Elghamery et al., 2000). Although shoot length increased in presence of NPs but the shoots were thin and with long inter nodal distance. This might be reason that reduction in shoot FW was observed. The presence of NPs in the culture media did not affect water holding capability of shoots so no significant change in DW was observed. On the other hand, the root FW and DW decreased by increasing NPs concentration. Presence of zinc NPs itself or dissoluted ion interferes with root biochemistry and physiology making variation in length and weight. A number of reports favor these results on root inhibition, i.e., radish, rape, ryegrass, lettuce, cucumber, and *Arabidopsis* (Lin and Xing, 2007, 2008; Lee et al., 2010). However, inhibitory effect may vary depending on plant species, size and shape of NPs; adherence potential to root surface, translocation capability from root to shoot and dissolution, and release of metallic ions in the surrounding medium (Franklin et al., 2007). Under field conditions wheat reduced biomass by ZnO NPs has been reported (Du et al., 2011). Heavy metals have been widely studied for their inhibitory effect on seed germination, growth, development, biochemical, and physiological processes (Prasad, 2004).

Presence of NPs in the medium induced oxidative stress both in shoots and roots, however, more in root due to direct interaction with NPs. Variations were observed in free radical scavenging response, total antioxidant response and reducing power potential and these parameters are found directly linked with non-enzymatic antioxidative molecules; phenolics and flavonoids. Both, phenolics and flavonoid contents significantly varied in shoots and roots. Few research focus on promotion of oxidative stress in living species on NPs exposure (Wilson et al., 2002; Sayes et al., 2005; Long et al., 2006). The photocatalytic activity of ZnO also involves in ROS response in organisms because of their band gap energy 3.37 eV (Ma et al., 2009, 2011) though band gap energy is not essential for generation of ROS (Navarro et al., 2008). Although zinc is important element for many metabolic processes but high concentration interferes photosynthesis (Chaney, 1993) and generates oxidative stress (Prasad et al., 1999). It is also important to consider that some NP may generate bioprotective effect against oxidant injury (Chen et al., 2006). Elevated ROS has been observed by ZnO NPs to *Allium cepa* plants (Kumari et al., 2011).

### Effect of ZnO NPs on Stem Explant Growth and Antioxidative Response

In the presence of ZnO NPs, stem explant responded differently depending upon concentration of NPs. The control produced green friable calli, while ZnO induced roots and shoots. AT lower concentration calli produced and some roots also induced from calli. While, at higher concentrations the rooting response was at greater extent. At 10 mg/L NPs few shoots also emerged that were green but watery. The induction of organ from calli let to reduction in fresh and DW. No report is available on application of NPs on *in vitro* culture of explant. The induction of roots can be explained in two ways; (i) function of zinc in biochemical process, and (ii) role of ROS. The acidic nature of MS medium may increase dissolution behavior of ZnO NPs into zinc ion. Where zinc ion functions as cofactor for several enzymes such as oxidases, dehydrogenases, anhydrases, and peroxidases (Hewitt, 1983) and plays role in regulating auxin synthesis, nitrogen metabolism, and cell multiplication (Tsui, 1948; Stanisławski, 1977; Shier, 1994). Cakmak et al. (1989) reported that bean plants provided with sufficient supply of Zn produced IAA as compared to Zn deficient plants. This role of zinc has also been observed in tomato and *Eucalyptus globulus* by applying auxin and Zn that enhanced root formation (Buczek, 1964; Schwambach et al., 2005). Although, zinc itself regulated the synthesis of endogenous plant hormones; presence of auxin also regulates local synthesis of cytokinin by controlling the expression of *adenosine phosphate*–*isopentenyltransferase* (*PsIPT*) gene, which encodes a key enzyme in cytokinin biosynthesis (Tanaka et al., 2006). Auxin and cytokinin also interact at metabolic level to control plant development and emergence of one part initiate to synthesize hormones for opposite one (Nordstrom et al., 2004). On the other hand, ROS generated in explants might also be involved in induction of roots from explant/callus. ROS works as signaling molecule at non-toxic level (Mittler et al., 2004). Furthermore, cellular proliferation upon accumulation of $O_{2}^{–}$ and differentiation upon elevated H_2_O_2_ levels have been reported, i.e., in zebra fish ROS trigger and execute a developmental process (Niethammer et al., 2009). Another aspect of ROS growth regulation involves superoxide, that affects root growth and root hair development (Foreman et al., 2003) through the regulation of calcium channels (Foreman et al., 2003; Jones et al., 2007). Recent reports demonstrated that the concentration of NPs and plant species have significant role defining the toxicity. Sosan et al. (2016) stated that nanoparticles trigger Ca^2+^ and ROS signaling at cellular level along with other complex physiological modifications at organism level. Furthermore, Hossain et al. (2016) analyzed expression of proteins harboring hormone metabolism, cell organization, signaling, and stress functionalities in soybean on nanoparticles exposure.

## Conclusion

In summary, we have experimentally demonstrated the effect of ZnO NPs concentration on the *B. nigra* seed germination and seedling growth. We have also observed that higher NPs concentration led to an increase in the antioxidative activities and non-enzymatic antioxidants. On the other hand, lower concentration of ZnO NPs resulted in the production of white thin roots with thick root hairs and somewhat shoots from stem explants of *B. nigra*. Moreover, the presence of NPs led to significant effect on total antioxidant and reducing power potential. Based on experimental results we believe that ZnO NPs may induce roots from explants cultured on appropriate medium that can be used for production of valuable secondary metabolites.

## Author Contributions

All authors listed, have made substantial, direct and intellectual contribution to the work, and approved it for publication.

## Conflict of Interest Statement

The authors declare that the research was conducted in the absence of any commercial or financial relationships that could be construed as a potential conflict of interest.
